# Advancing tree genomics to future proof next generation orchard production

**DOI:** 10.3389/fpls.2023.1321555

**Published:** 2024-01-19

**Authors:** Stephanie C. Kerr, Saiyara Shehnaz, Lucky Paudel, Mekaladevi S. Manivannan, Lindsay M. Shaw, Amanda Johnson, Jose Teodoro J. Velasquez, Miloš Tanurdžić, Christopher I. Cazzonelli, Erika Varkonyi-Gasic, Peter J. Prentis

**Affiliations:** ^1^ School of Biology and Environmental Science, Queensland University of Technology (QUT), Brisbane, QLD, Australia; ^2^ Centre for Agriculture and the Bioeconomy, Queensland University of Technology (QUT), Brisbane, QLD, Australia; ^3^ School of Chemistry and Molecular Biosciences, The University of Queensland, Brisbane, QLD, Australia; ^4^ Hawkesbury Institute for the Environment, Western Sydney University, Penrith, NSW, Australia; ^5^ Queensland Alliance for Agriculture and Food Innovation, The University of Queensland, Brisbane, QLD, Australia; ^6^ School of Agriculture and Food Sustainability, The University of Queensland, Brisbane, QLD, Australia; ^7^ The New Zealand Institute for Plant and Food Research Limited, Auckland, New Zealand

**Keywords:** tree crops, architecture, precocity, flowering, dormancy, alternate bearing, genomics

## Abstract

The challenges facing tree orchard production in the coming years will be largely driven by changes in the climate affecting the sustainability of farming practices in specific geographical regions. Identifying key traits that enable tree crops to modify their growth to varying environmental conditions and taking advantage of new crop improvement opportunities and technologies will ensure the tree crop industry remains viable and profitable into the future. In this review article we 1) outline climate and sustainability challenges relevant to horticultural tree crop industries, 2) describe key tree crop traits targeted for improvement in agroecosystem productivity and resilience to environmental change, and 3) discuss existing and emerging genomic technologies that provide opportunities for industries to future proof the next generation of orchards.

## Introduction

Climate change is one of the major challenges of the 21^st^ century. The Intergovernmental Panel on Climate Change (IPCC, 2023) predicts an increase in average global temperatures between 1.4 and 4.4°C by the end of this century. Increased global temperatures are predicted to have vast flow-on effects on global climate and local weather systems leading to more extreme and unpredictable temperatures, as well as increased extreme rainfall, drought, storm and fire events (IPCC, 2023). Many of these impacts are already being experienced around the world, with NASA declaring July 2023 the hottest month on record fuelling heat waves, wildfires, and floods across the Northern Hemisphere. These changes will affect every tree crop industry and region to varying degrees. Temperate tree crops are at particular risk due to their high reliance on seasonal variations in temperature for processes such as bud dormancy and flowering (Campoy et al., 2011), and pollen development and fruit set (Irenaeus and Mitra, 2014), which all in turn impact yield. Subtropical tree crops are also not immune to the effects of climate change with many reliant on cold winter temperatures to induce flowering (Wilkie et al., 2008), and temperature also significantly impacting pollen and fruit development (Dinesh and Reddy, 2012).

Another challenge facing humanity this century is increasing population size and demand for food. Taagepera and Nemčok (2023) predict the global population to increase to a peak of 11.2 ± 1.5 billion by the end of this century. Population increases during the 20^th^ century were offset by the Green Revolution, a doubling in production of high-yielding varieties of several cereal crops (Khush, 1999). However, population growth since the 1990s has outstripped the rate of growth in food production requiring new strategies to increase cereal crop yield. Tree crops will require a more substantial increase in yield and reliability of yield as the Green Revolution gains in cereal crop productivity have not been implemented in tree crops. Adding to this challenge, the effects of climate change on yield are also uncertain, with changes in temperature, rainfall patterns and CO_2_ levels likely to significantly influence crop productivity (Aydinalp and Cresser, 2008).

One innovative change that is being implemented in some tree crop industries, e.g. olives (*Olea europaea*) in Europe (Lo Bianco et al., 2021), is the transition to higher density orchard plantings. This transition requires the use of small trees with low shoot vigour and, ideally, precocious flowering (i.e., flowering at a younger age). This is often achieved through orchard management but can also be achieved using varieties and/or rootstocks that confer these traits. However, some tree crops like avocado (*Persea americana*) and macadamia (*Macadamia integrifolia*, *Macadamia tetraphylla*, and hybrids) lack available varieties or rootstocks with these traits, and many horticultural industries have not made substantive use of available varieties and/or rootstocks. Planting at higher densities gives the potential for higher yield per land as seen in olive orchards (Sobreiro et al., 2023), however, this may not be applicable to all tree crops (Haque and Sakimin, 2022). The benefits of higher density plantings are increased if trees flower and produce high yield at a younger age as this improves the return on investment and allows for faster turn-around of plantings and quicker introduction of newer, improved varieties. Another advantage of higher density plantings is that they are more amenable to implementation of automation/robotics for crop management and harvest. This is especially pertinent in countries with high wages and labour scarcity (Rutledge and Taylor, 2023) that often cannot compete on a cost level with countries with much lower wages. The use of automation can dramatically reduce the ongoing costs associated with crop management and harvest to make these industries remain competitive on an international standing.

Although responding to these challenges will require a multi-pronged approach, it is imperative that genomic technologies are harnessed to complement traditional breeding efforts to enhance and accelerate the process. The advent of low-cost genome sequencing and associated increase in high quality genome assemblies and ability to re-sequence large-scale germplasm and hybrid populations (Okemo et al., 2022) has dramatically advanced the implementation of genomic approaches into breeding programs. In particular, high-throughput sequencing enables the use of genome-wide association studies (GWAS) and marker-assisted selection (MAS) for high-throughput assessment of breeding populations to identify preferred parental crosses and filter progeny for traits of interest. However, as many tree crops are highly heterozygous outcrossers (Miller and Gross, 2011), traditional crossing and selection remains challenging as many desirable traits can be lost during crossing. The ability to genetically manipulate crops for trait improvement using newer gene editing technologies such as CRISPR holds the promise of genetically engineering traits without the need for genetically modified (GM) cultivars (Hu and Gao, 2023). These technologies allow for the genetic manipulation of individual genes while maintaining other desirable traits, although, as we will discuss later, many challenges remain. And while these approaches have been extensively used in many cereal crops, their use in most tree crops has been relatively unexplored and underutilised to date.

**Figure 1:**
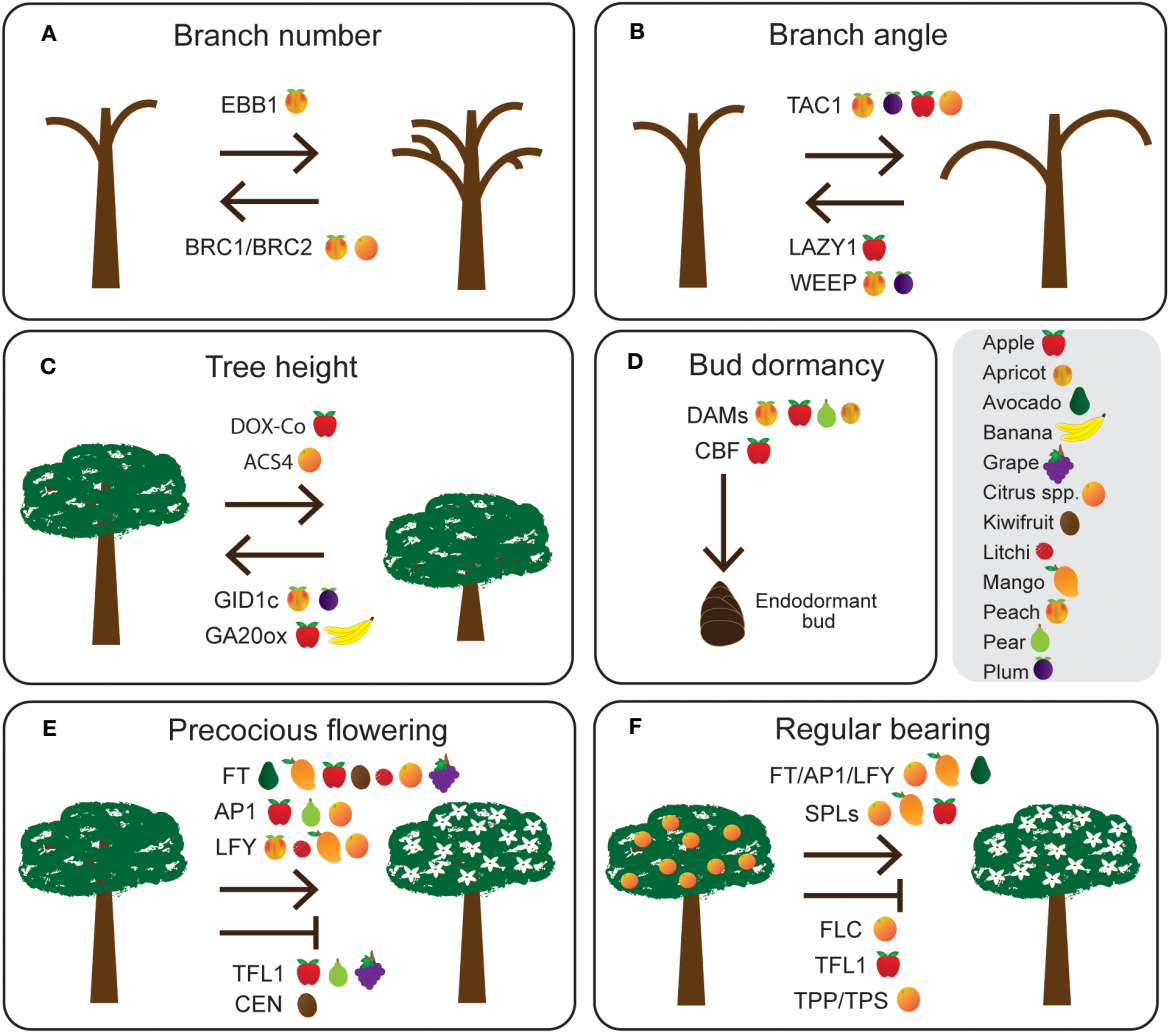
Potential gene targets controlling agronomically important traits such as **(A)** branch number, **(B)** branch angle, **(C)** tree height, **(D)** bud dormancy, **(E)** precocious flowering, and **(F)** regular bearing in tree crops. Species (grey box) with evidence of gene function or gene expression are indicated after each gene name.

**Figure 2:**
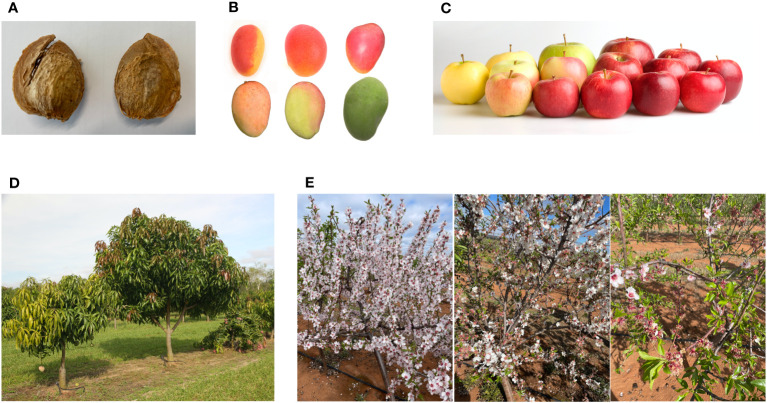
Pictures of several traits targeted for improvement in various tree crop breeding programs. **(A)** Nutshell seal in almond; The nut on the left demonstrates a variety with poor nutshell seal which increases risk of insect damage and diseases, while the nut on the right demonstrates a variety with good nutshell seal. **(B)** Fruit blush in mango; Consumer preference for fruit blush differs between countries and is mainly dependent on the main varieties available. In Australia a consistent red fruit blush (top) is preferred over inconsistent or no fruit blush (bottom). **(C)** Fruit characteristics in apple; fruit characteristics such as fruit peel colour and fruit size are also dependent on consumer preference. **(D)** Shoot architecture in mango; The tree on the right is a conventional mango tree with high vigour, while the tree on the left has low vigour with a small shoot architecture preferable for high density plantings. **(E)** Flowering time in almond; Almond varieties can be early (left), mid (middle) or late (right) flowering depending on their chill requirements during endodormancy, with ideal timing of flowering dependent on location and local climate.

In this review we describe several key traits and genetic targets (**Figure 1**) that are a focus for genetic improvement in tree crops in response to the challenges raised above. First, we explore the intensification of crop density through the modification of different aspects of tree architecture, including branching number, branching angle, and tree height. Next, we discuss bud dormancy in deciduous trees and how changes in temperature due to climate change may affect this important biological process. Following this we consider early, or precocious, flowering to accelerate tree crop breeding. And finally, we explore alternate bearing, a phenomenon experienced in many tree crops that influences yield predictability. While these traits are key targets for genetic improvement, there are many other important traits that are being targeted for improvement by breeding programs (see examples in **Figure 2**). In this review we also discuss existing and emerging genomic technologies that can be utilised for accelerating genetic improvement in tree crops. These rapid and precise genome-enabled technologies first require an understanding of the genetic basis of important traits in tree crops to inform marker assisted and genomic selection models or genetic manipulation of the tree crops through transgenics or gene editing.

## Modification of crop density and tree architecture

Due to their extended lifespan, trees are in constant need of modifying their growth in response to the surrounding environment (Cooke et al., 2012). Tree architecture is an excellent example of this developmental plasticity, which is maintained via the regulation of axillary bud dormancy (Wang M. et al., 2019). Following establishment, axillary buds are maintained in a dormant state through a combination of various environmental and endogenous signals acting to ensure dormancy breaks at an appropriate time resulting in bud outgrowth and, if appropriate signals persist, in shoot branch formation (Wang M. et al., 2019). This high reliance of plant plasticity on environmental cues is increasingly becoming an issue in the face of rapidly changing, unreliable climatic conditions. Research focused on deciphering the genetic and molecular components/mechanisms regulating traits like shoot architecture and flowering is therefore critical to our ability to mitigate the impact of climate change on tree growth and productivity. Here, we will outline current knowledge of several key genetic components in tree crops regulating three specific shoot architecture traits: branching number, branching angle, and plant height. For horticultural crops, trees with shorter or less branches, and semi-dwarf phenotypes may be the ideal architecture allowing for higher planting density and reduced mechanical intervention like pruning (Scorza, 2005; Hollender and Dardick, 2015).

Shoot architecture has been a key target of domestication in many crops, with reduced branch number being an important component of the ideal shoot architecture (**Figure 1A**). In crops such as maize (*Zea mays* spp. *mays*), a decrease in tiller number correlates with yield increases (Doebley et al., 1997). The wild ancestor of maize (*Zea mays* spp. *parviglumis*) has an increased axillary branching phenotype (Doebley et al., 1995) and the gene controlling this trait was identified by Doebley et al. (1997) as *TEOSINTE BRANCHED1* (*TB1*) which belongs to the TEOSINTE BRANCHED1, CYCLOIDEA, PROLIFERATING CELL FACTOR (TCP) family of transcription factors. Since then, orthologs have been identified and studied in fruit trees such as peach (*Prunus persica*) where overexpression of *PpTCP18*, a TB1/BRANCHED1 (BRC1) ortholog, in *Arabidopsis thaliana* caused a decrease in rosette branch numbers (Wang X. et al., 2023). Interestingly, in Carrizo citrange (*Citrus sinensis* x *Poncirus trifoliata*) the BRC1 ortholog THORN IDENTITY1 (TI1) acts partially redundantly with the BRC2 ortholog TI2 to control shoot branching alongside thorn identity (Zhang et al., 2020).

Shoot branch angle is another important aspect affecting overall shoot architecture which can lead to trees having an upright and erect architecture, termed pillar or broomy, or a spread apart architecture, termed weeping (**Figure 1B**). LAZY1 acts as a negative regulator of the polar auxin transport (PAT) to decrease the response to gravity, and loss-of-function mutations in rice promote a branching phenotype that is more spread apart (Li et al., 2007; Yoshihara and Iino, 2007). In apple (*Malus domestica*), the *MdLAZY1A-W* allele has a single nucleotide substitution that changes the amino acid from leucine to proline resulting in a dominant weeping phenotype (Dougherty et al., 2023). In contrast, an in-frame stop codon mutation in *LAZY1* in silver birch (*Betula pendula*) leads to a recessive weeping phenotype (Salojärvi et al., 2017). *LAZY1* belongs to the IGT gene family alongside another regulator of shoot branch angle, *TILLER ANGLE CONTROL1* (*TAC1*). Mutations in rice (*Oryza sativa*) *OsTAC1* result in a more compact phenotype (Yu et al., 2007; Jiang et al., 2012). The gene has since been identified in other species including the fruit trees peach (Dardick et al., 2013), plum (*Prunus domestica*; Hollender et al., 2018b), apple (Li et al., 2022), and sweet orange (*Citrus sinensis*; Dutt et al., 2022). In peach, the likely cause of the pillar phenotype in the ‘Italian pillar’ cultivar is an insertion causing a premature stop codon in *PpeTAC1* (Dardick et al., 2013). Overexpression of *PpeTAC1* in plum increased the branch angle, while RNA interference (RNAi) of *PdoTAC1* in plum reduced the branch angle leading to a more upright architecture (Hollender et al., 2018b). Similarly, sweet orange *CsTAC1* knockout transgenic lines displayed a more compact branch angle, and this was associated with low auxin levels and upregulation of *CsBRC1* expression, following a similar expression pattern to Arabidopsis (Dutt et al., 2022). Another regulator of branching orientation identified in peach is the *WEEP* gene, encoding a sterile alpha motif (SAM) domain protein. The shoots on WEEP trees initially grow upwards but, after reaching a certain height (20 cm), start exhibiting a weeping phenotype (Hollender et al., 2018a). Grafting of buds from WEEP trees to wild-type rootstocks did not rescue the weeping phenotype of the scion suggesting that there is no requirement for a mobile or systemic signal for the weeping phenotype. Transcriptomic analysis showed differential expression of auxin-related genes like *AUXIN/INDOLE-3-ACETIC ACID* (*Aux*/*IAA*s) and *AUXIN RESPONSE FACTOR*s (*ARF*s) indicating a role for auxin in the gravitropic response (Hollender et al., 2018a). RNAi of the *WEEP* gene in plum resulted in a weeping phenotype (Hollender et al., 2018a), showing conserved function of WEEP in a closely related tree species.

Plant height is another key trait that influences overall shoot architecture (**Figure 1C**). In the 1960s, a specific type of architecture with short internodes and fewer axillary branches was observed in the mutant apple McIntosh Wijcik (Fisher, 1969). Further research identified a dominant 2-oxoglutarate-dependent dioxygenase (DOX) gene, *Columnar* (*Co*) or *MdDOX-Co,* as the likely candidate responsible for the mutant phenotype (Lapins, 1976; Okada et al., 2016). Overexpression of *MdDOX-Co* in tobacco (*Nicotiana tabacum*) resulted in a dwarfed phenotype with a shorter main stem and internodes (Okada et al., 2020). This was associated with decreased endogenous bioactive gibberellin (GA) levels and exogenous application of GA was able to rescue the dwarfed phenotype (Okada et al., 2020). Overexpressing *MdDOX-Co* in Arabidopsis led to the discovery that it regulates 12-hydroxylation of GA_12_, a precursor in the GA biosynthesis pathway, to reduce active GA levels resulting in the dwarf phenotype (Watanabe et al., 2021). Similar to the McIntosh Wijcik mutant, the peach *dw* mutants have a dwarfing phenotype associated with short internodes (Scorza, 1984). Mapping analysis identified a GA receptor gene *GIBBERELLIN INSENSITIVE DWARF1c* (*GID1c*) as the likely cause of the *dw* trait, and RNAi-induced silencing of *PpeGID1c* in plum led to varying degrees of dwarfing (Hollender et al., 2016). Rice breeding has for many years employed the use of the semidwarf (*sd-1*) allele, caused by a defective GA biosynthetic gene *GIBBERELLIN 20-OXIDASE* (*GA20ox*) to decrease overall size while increasing yield (Spielmeyer et al., 2002). Multiple GA biosynthesis genes were identified as differentially expressed between the ‘Williams’ banana (*Musa acuminata*) 8818-1 wild-type cultivar and its dwarf counterpart (Chen et al., 2016), and there are already cases of successfully using *GA20ox* gene(s) as a target to produce dwarf fruit varieties, such as via suppression of *MpGA20ox1* in apple (Bulley et al., 2005) and via CRISPR-based silencing of five *MaGA20ox2* genes in banana (Shao et al., 2020). Ethylene is another plant hormone that has been associated with plant height regulation. Overexpression of the lemon (*Citrus limon*) gene *1-AMINOCYCLOPROPANE-1-CARBOXYLIC ACID SYNTHASE 4* (*CiACS4*), involved in the ethylene biosynthesis pathway, in tobacco and lemon resulted in dwarf plants (Chu et al., 2023). *CiACS4* was found to be directly upregulated by an ethylene response factor, CiERF023, and the CiACS4 protein also interacted with another ethylene response factor, CiERF3, to directly regulate the GA biosynthesis genes *CiGa20ox1/2*.

Here, we have discussed three main routes to shoot architecture manipulation: shoot branching numbers, branching angle, and/or overall plant height. The genetic regulation of these traits shows high levels of conservation between divergent plant species, thus providing several potential targets for improving architecture in other tree crops. Efforts to implement breeding and genomics-enabled approaches to alter these traits will provide significant inroads to achieving ideal tree architecture with maximised yield.

## Regulation of bud dormancy in deciduous trees

Deciduous trees that grow in temperate regions experience fluctuating temperature and daylength due to annual seasonal changes and have evolved to adapt to these environmental conditions by modulating their annual cycle of active growth and growth cessation. In deciduous trees, both terminal and axillary buds (whether vegetative or reproductive) temporarily stop visible growth during winter and only resume growth and flowering when the season is favourable (Singh et al., 2017). Growth cessation, bud set, cold acclimatisation and establishment of bud dormancy occur in sequential order with some overlap (Singh et al., 2017) creating difficulty not only in delineating where growth cessation ends and dormancy starts, but also in differentiating phenotypes and genetic mechanisms that are associated with these processes. In the state of deepest dormancy commonly referred to as endodormancy (**Figure 1D**; Lang et al., 1985), growth is repressed and cannot be initiated by growth-promoting conditions until the buds accumulate a certain number of hours of cold temperature known as the chilling requirement. Although this is a gradual process (Cooke et al., 2012), the transition to the ability of the plant to resume growth in permissive conditions is often referred to as ecodormancy (Lang et al., 1985). Release from dormancy is followed by budbreak, primarily driven by accumulation of heat (Alburquerque et al., 2008). Thus, dormancy is an important physiological and adaptive process that helps plant buds survive harsh winter temperatures and is an important determinant of yield in deciduous tree crops.

**Figure 3:**
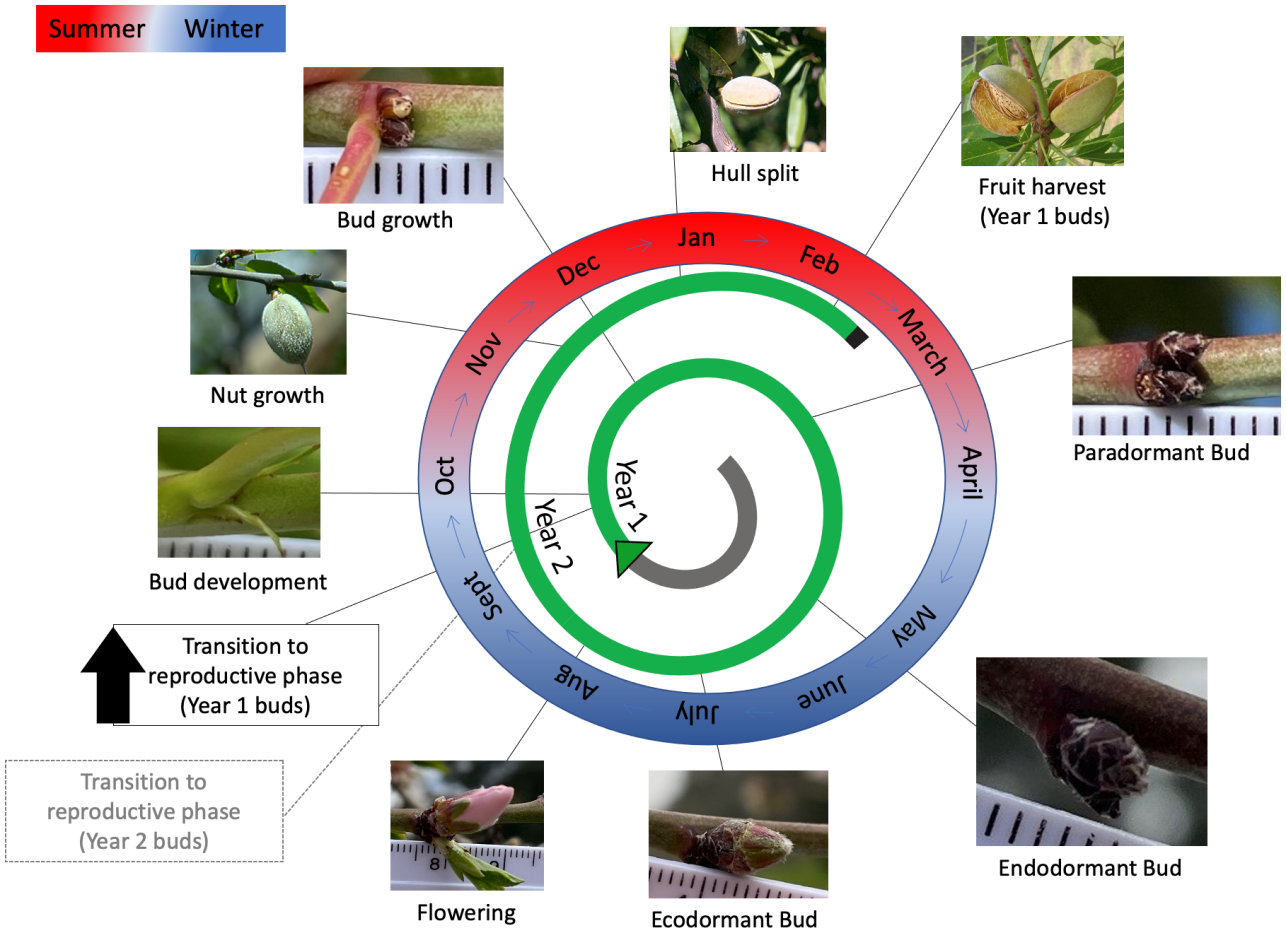
Phenology for the almond (*Prunus dulcis*) cultivar ‘Nonpareil’ grown in South Australia. The transition to flowering of new buds formed takes place in September 30-40 days after flowering. Buds are very small at this stage; however, the green, scale-like structures are visible at the nodes. The bud grows throughout summer. In the latter half of summer, buds are completely covered by layers of dark-brown scales. During the end of April and beginning of May, all almond leaves senesce, which signals the start of endodormancy. Buds accumulate chilling during May, June, and July. Once the chilling is complete, the bud enters ecodormancy. Ecodormant buds swell after some heat accumulation, then grow to develop flowers at the beginning of August. The fertilised ovaries grow throughout spring and summer to develop into fruit nuts. Once nut growth is complete, the hull starts to split in January. In February, the almond shell starts to desiccate, and the hull split widens indicating that the nuts are ready for harvest. The entire process from bud formation to harvest takes about 1.5 years; therefore, between September and February, almond trees have young buds from the current season and developing nuts from previous seasons’ buds.

Chilling and heat requirements are specific to the plant species and genotype, and reflect the long-term adaptation to specific growing conditions in their native or cultivated regions (Luedeling, 2012). For example, the specific phenology of the almond (*Prunus dulcis*) cultivar ‘Nonpareil’ grown in South Australia is depicted in **Figure 3**. Delayed budbreak and low yields arising as a consequence of reduced winter chilling in the warming climate are posing a global threat to agriculture and food security (Luedeling, 2012; Atkinson et al., 2013). For this reason, tree phenology has been a subject of extensive research, with significant progress made in understanding the environmental, physiological, genetic, epigenetic and hormonal regulation of dormancy (Lloret et al., 2017; Beauvieux et al., 2018; Liu and Sherif, 2019; Fadón et al., 2020; Pan et al., 2021; Yamane et al., 2021; Yang Q. et al., 2021; Nilsson, 2022). Not only do we need in-depth understanding of the molecular mechanisms regulating dormancy, but also taking advantage of next generation molecular techniques and translating these advancements to the orchard is crucial for plant biologists to develop new and improved cultivars better suited to the new climate dynamics.

In Rosaceous plants like almond, peach, plum, pear (*Pyrus* spp.) and apple, *DORMANCY ASSOCIATED MADS-BOX* (*DAM*s) transcription factors are central regulators of bud dormancy during winter chilling (**Figure 1D**). *DAM*s were first identified in a natural mutant peach cultivar *evergrowing*, which fails to enter dormancy during winter (Bielenberg et al., 2008). Sequencing revealed six genes that were either partially or fully deleted in the mutant compared to wild-type peach and were termed as *DAMs* (Bielenberg et al., 2008). Various studies in apple (Wu et al., 2017), Japanese pear (*Pyrus pyrifolia*; Saito et al., 2013; Niu et al., 2016; Tuan et al., 2017) and Japanese apricot (*Prunus mume*; Yamane et al., 2019) have shown that overexpressing *DAM*s prolongs dormancy in these trees, and silencing or mutagenesis prevents dormancy in hybrid aspen (*Populus tremula* x *P. tremuloides*; Singh et al., 2019) and apple (Moser et al., 2020; Wu et al., 2021). A recent study by Falavigna et al. (2021) explored the gene regulatory networks controlled by DAMs during the dormancy cycle in apple in response to environmental cues and hormonal signalling pathways. Transcriptomic studies in various other species including WT-717 poplar hybrid, Japanese pear and raspberry (*Rubus idaeus*) show that *DAM*s, as well as important genes involved in abscisic acid (ABA) and GA synthesis and catabolism differentially accumulate in buds transitioning from growth cessation through the different stages of dormancy to growth resumption in spring (Mazzitelli et al., 2007; Ruttink et al., 2007; Niu et al., 2016). ABA has been shown to control dormancy by inhibiting genes involved in cell proliferation and growth, and activating catabolism genes of other hormones such as GAs (Yang Q. et al., 2023). However, the exact signalling cascades by which DAMs trigger ABA and GA pathways in response to environment are still obscure.

While DAMs play an important, conserved role in regulating bud dormancy in many temperate crops, other genes are also likely to play an important role in this process. For example, in apple the major quantitative trait locus (QTL) controlling bud dormancy does not contain any *DAM* genes suggesting other gene(s) are regulating bud dormancy in apple (Allard et al., 2016). The *C-REPEAT BINDING FACTOR1* (*PpCBF1*) gene has been shown to play a role in bud dormancy when overexpressed in apple (Wisniewski et al., 2015). While *CBF* and other genes likely play a role in bud dormancy, the DAMs are clearly critical regulators of the onset and release of bud dormancy in Rosaceae species. Delving deeper into the downstream targets regulated by DAMs can reveal new targets towards modifying bud dormancy according to the geographical climates of orchard production.

## Promoting earlier flowering (Precocity)

A critical developmental time in the life cycle of a tree is the vegetative to reproductive phase change, during which the tree attains the ability to flower and produce seeds. When compared to an annual plant, which can flower, produce seeds, and complete its life cycle within one year, a woody perennial tree will often not reach reproductive maturity for several years or even decades (Hackett, 1985). Following the first onset of flowering, trees flower annually in response to environmental signals but commit only some of the meristems to flowering, thereby maintaining the polycarpic growth habit (Amasino, 2009). The delayed maturity, long life span, and polycarpic growth of trees require a complex regulatory network to regulate the timing of developmental transitions and synchronise environmental signals with phenological events to ensure survival and successful reproduction (Brunner et al., 2017). While flowering gene homologs from woody perennials often show a conserved role in regulation of flowering in model annuals such as Arabidopsis and tobacco, functional studies in trees reveal shared and distinct functional aspects, highlighting the diverse roles of paralogs (Hsu et al., 2011; Sheng et al., 2022) and the pleiotropic effects of flowering genes on growth (André et al., 2022; Sheng et al., 2023), organ identity (Zhang et al., 2021), and phenology in trees (Ding and Nilsson, 2016). Therefore, targeting specific genes to reduce the years to reproduction in trees is a complex task that depends on the species in question. However, the types of genes that could be targeted to accelerate reproductive maturation are those regulating flowering time and floral meristem identity (**Figure 1E**).

Members of the phosphatidylethanolamine–binding protein (PEBP) family are crucial for floral transition. The divergence of PEBP genes into *FLOWERING LOCUS T* (*FT)* (predominantly activators of flowering) and *TERMINAL FLOWER1* (*TFL1*)/*CENTRORADIALIS* (*CEN*) (predominantly repressors of flowering) (Wickland and Hanzawa, 2015) subgroups in angiosperms was critical for the reproductive success of flowering plants (Shalit et al., 2009; Karlgren et al., 2011). Further divergence through gene duplication events, followed by neo- and sub-functionalisation, gave rise to FT-like regulators implicated to have broader functions in plant development and adaptation (Pin and Nilsson, 2012). It is important to note that the specific functions and regulatory mechanisms of FT in perennial fruit trees can vary between different species and cultivars within the same species, but FT has generally maintained its role as an inducer of flowering. Early flowering can be induced in Arabidopsis by overexpressing *FT* orthologs from many diverse tree crops such as avocado (Ziv et al., 2014), mango (*Mangifera indica*; Fan et al., 2020), apple (Kotoda et al., 2010), kiwifruit (*Actinidia chinensis*; Voogd et al., 2017), and litchi (*Litchi chinensis*; Ding et al., 2015); while constitutive ectopic expression of *FT* promotes early flowering in tree crops such as apple (Kotoda et al., 2010), trifoliate orange (*Poncirus trifoliata*; Endo et al., 2005), and kiwifruit (Voogd et al., 2017). However, in some instances, overexpression of *FT* causes *in vitro* and abnormal flowering such as that observed in apple (Kotoda et al., 2010) and kiwifruit (Voogd et al., 2017; Moss et al., 2018). More recently, it was shown that FT chimeric proteins induce floral precocity without detrimental effects in citrus (Sinn et al., 2021) and kiwifruit (Herath et al., 2023) species, suggesting that this approach may be universally applicable for woody perennial crops.

The concept of grafting on rootstocks engineered to overproduce FT to accelerate flowering of the scion has been explored as means of flowering control in horticultural and agricultural practices. However, the movement of FT and its ability to promote flowering in trees is still under debate (Putterill and Varkonyi-Gasic, 2016), with reports of movement across the graft union without precocity observed in juvenile apple, trifoliate orange, hybrid aspen or WT-717 poplar hybrid scions (Zhang et al., 2010; Wenzel et al., 2013; Freiman et al., 2015; Miskolczi et al., 2019; Wu et al., 2022), while precocious flowering was observed in Carrizo citrange (Soares et al., 2020) and *Jatropha curcas* scions (Ye et al., 2014). In various *Jatropha* spp., the distance between the graft union and bud is important for floral induction and increasing this distance can lead to a lower frequency of flowering (Tang et al., 2022). Therefore, grafting approaches may be dependent on many variables including the plant species, plant/scion size, as well as the *FT* gene and its regulatory promoter.

Plant viral vectors have also been used to either express *FT* for virus-induced flowering or silence *TFL1* via virus-induced gene silencing (VIGS), and these approaches have induced early flowering in apple, Japanese pear, European pear (*Pyrus communis*), and *Vitis* spp. (Sasaki et al., 2011; Yamagishi et al., 2014; Velázquez et al., 2016; Yamagishi et al., 2016; Maeda et al., 2020). RNA silencing of *TFL1*/*CEN* has also induced early flowering in apple (Kotoda et al., 2006), WT-717 poplar hybrid (Mohamed et al., 2010) and European pear (Freiman et al., 2012). Recently, CRISPR/Cas9-mediated mutagenesis of one or two *CEN* genes induced fast flowering in several kiwifruit species (Varkonyi‐Gasic et al., 2019; Varkonyi-Gasic et al., 2021; Herath et al., 2023) and editing of *TFL1* induced early flowering in apple and European pear (Charrier et al., 2019). However, editing of a blueberry (*Vaccinium corymbosum*) *CEN* gene did not result in precocious flowering (Omori et al., 2021), suggesting that some TFL1/CEN genes perform different roles. Indeed, mutagenesis of a kiwifruit *BROTHER OF FT AND TFL1* (*BFT*) prevented the establishment of dormancy without affecting flowering (Herath et al., 2022), and in Carrizo citrange loss of *CsCEN* resulted in the conversion of axillary meristems to thorns, while ectopic *CsCEN* expression converted thorns to axillary meristems (Zhang et al., 2021).

Floral meristem identity genes perform as key outputs of FT-mediated floral transition or FT-independent flowering pathways. They include MADS-box proteins such as APETALA1 (AP1) and SUPPRESSOR OF OVEREXPRESSION OF CONSTANS1 (SOC1), necessary to initiate and maintain reproductive development, and LEAFY (LFY) acting as a pioneer transcription factor reprogramming vegetative meristematic cells into an inflorescence (Jin et al., 2021; Lai et al., 2021). *LFY* homologs from trees, including peach, litchi and mango, can induce flowering when overexpressed in annual models (An et al., 2012; Ding et al., 2018; Wang Y. et al., 2022). Ectopic overexpression of *LFY* also promoted flowering in Carrizo citrange (Peña et al., 2001), yet there were some inconsistencies in floral induction between various *Populus* hybrids that displayed altered flower development as well as sexual differentiation (Weigel and Nilsson, 1995; Rottmann et al., 2000). MADS-box genes have been important targets for selection during crop domestication and improvement as they play pivotal roles in every aspect of plant reproductive development, controlling flowering time, inflorescence architecture, determination of floral meristem and floral organ identity, and seed development (Schilling et al., 2018). The ability of *AP1* homologs to induce flowering in a range of woody perennials, e.g. Carrizo citrange, silver birch and apple (Peña et al., 2001; Elo et al., 2007; Flachowsky et al., 2007), has been adopted for rapid cycle breeding approaches in apple and European pear (Flachowsky et al., 2011; Tomes et al., 2023). However, functional studies in hybrid aspen revealed that, like *FT*, homologs of MADS-box genes controlling the regulation of flowering time in annual plants have a role in control of vegetative phenology in trees (Azeez et al., 2014; Ding and Nilsson, 2016).

**Figure 4:**
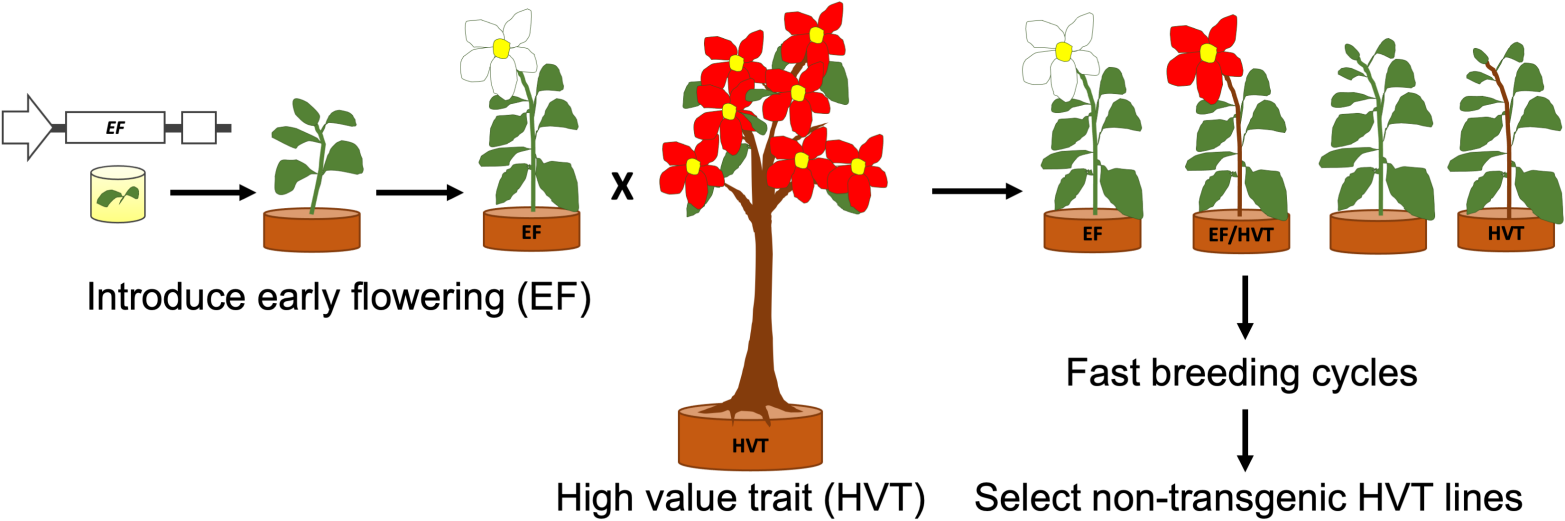
A schematic diagram representing fast breeding of tree crops using early flowering (EF) transgenic lines to introduce a high value trait (HVT). Half of the progeny is EF, with half of those also carrying the HVT gene(s). Non-transgenic lines are selected after a desired number of breeding cycles.

This section has highlighted several key genes and gene families that offer valuable tools for shaping and improving developmental and reproductive traits in crops. Their manipulation through targeted breeding approaches can contribute to the accelerated development of new crop varieties with enhanced characteristics (as demonstrated in **Figure 4**), ultimately benefiting horticultural food production.

## Managing alternate bearing for more consistent yields

Alternate bearing, also known as biennial bearing, is a significant issue faced by the horticultural industry and has been reported to affect mango, cranberry (*Vaccinium macrocarpon*), avocado, olive, apple, European pear, plum, apricot (*Prunus armeniaca*), coffee (*Coffea arabica*), *Citrus* spp., litchi and nut producing trees (Monselise and Goldschmidt, 1982). Alternate bearing is initiated by an abnormally heavy fruit load one year, decreasing the ability of the tree to undergo floral initiation and resulting in a light fruit load the following season. This pattern of alternating high then low crop production years can differ in intensity between cultivars within a species, and can also be strongly influenced by environmental factors (Monselise and Goldschmidt, 1982). These inconsistent patterns of bearing and yield have major implications for growers and for responding to the need for reliable food sources into the future.

Studies have demonstrated that fruit load affects flowering the following season by repressing the expression of genes involved in floral initiation (**Figure 1F**). *FT*, *AP1* and *LFY* are shown to be highly expressed in the absence of fruit and have minimal to no expression in the presence of a heavy crop load in avocado (Ziv et al., 2014), mango (Nakagawa et al., 2012; Das et al., 2019), and ‘Moncada’ mandarin (*Citrus clementina* x Kara mandarin (*C. unshiu* x *C. nobilis*); Muñoz-Fambuena et al., 2011). In avocado, expression of floral initiation genes was suppressed in shoots with high fruit load, and the transition from vegetative to reproductive structure was completely repressed with young leaves appearing instead (Ziv et al., 2014). In *Citrus* spp., *CiFT2* expression is downregulated in trees with high fruit load by CcMADS19, an ortholog of the floral repressor FLOWERING LOCUS C (FLC), while in trees with a low fruit load, low *CcMADS19* expression results in an increase in *CiFT2* expression (Agustí et al., 2020). Expression of *CcMADS19* itself was shown to be correlated with epigenetic changes (Agustí et al., 2020). Fluctuations in activity of *SQUAMOSA PROMOTER BINDING PROTEIN-LIKE* (*SPL*) genes have also been observed in response to fruit load in mandarin (*Citrus reticulata*; Shalom et al., 2012), mango (Sharma et al., 2020), and apple (Guitton et al., 2016).

Studies in ‘Murcott’ mandarin (*Citrus reticulata* x *C. sinensis*) and olive have shown the possible involvement of auxin (IAA) in alternate bearing, with auxin transmitting a heavy-fruit-load signal to the shoot apical meristem to repress floral initiation, and thus playing a role in determining whether vegetative or floral development proceeds (Haim et al., 2021). Peaks in transcript levels of IAA transporter genes were also associated with the induction of the flowering-inhibition signal (Haim et al., 2021). It has been shown that auxin induces the biosynthesis of GA during fruit set in tomato (*Solanum lycopersicum*; de Jong et al., 2009; Hu et al., 2018) and woodland strawberry (*Fragaria vesca*; Liao et al., 2018), and GA has been widely shown to promote vegetative growth and inhibit flower formation in tree crops. Heavy fruit load increases GA levels in ‘Satsuma’ mandarin (*Citrus unshiu*) leaves (Koshita et al., 1999), and fruit load affects the expression patterns of GA metabolism genes in mango (Nakagawa et al., 2012; Sharma et al., 2020). Exogenous application of GA also leads to a significant increase in *TFL1* expression in apple (Haberman et al., 2016) and reduced *FT* expression in ‘Orri’ mandarin (*Citrus reticulata* x *C. temple*; Goldberg-Moeller et al., 2013), apple (Elsysy and Hirst, 2019), and mango (Krishna et al., 2017). Therefore, in fruit trees, auxin likely plays a role in mediating fruit-load inhibition of flowering by increasing GA levels to regulate expression of both floral promoter and repressor genes.

Another important factor regulating alternate bearing is resource availability, in particular, carbohydrate levels. Competition for carbohydrates between developing fruit and nearby apical buds leads to depletion of carbon levels and reduced cellular activity in vegetative meristems, blocking the onset of floral development and potentially inducing bud dormancy (Tarancón et al., 2017; Martín-Fontecha et al., 2018). Tree starch content is significantly depleted by a heavy crop load resulting in fewer flowers and a lower crop load the following year (Nakagawa et al., 2012). Sugars are increasingly recognised as important signalling molecules (Fichtner and Lunn, 2021) with several sugar signalling pathways associated with bud outgrowth. In mandarin, buds on branches with high fruit-load showed induction of the trehalose metabolism enzymes, *TREHALOSE PHOSPHATE PHOSPHATASE* (*TPP*) and *TREHALOSE PHOSPHATE SYNTHASE* (*TPS*), which play a role in flowering in model species (van Dijken et al., 2004), suggesting a possible role for trehalose and/or its biosynthetic pathway in the transition to flowering (Shalom et al., 2012). The promotion or repression of flowering may be regulated by changes in sugar signalling which reflects changes in resource availability i.e., when many fruit are present, buds are deprived of photoassimilates, preventing flower bud formation.

By improving our understanding of the molecular regulation of alternate bearing we can continue to develop and optimise targeted tree management strategies, such as plant growth regulator application, nutritional management and regulating carbohydrate depletion, to ensure the consistent promotion of flowering and fruit development. Additionally, the significant variation in susceptibility to alternate bearing observed between cultivars offers potential targets for genetic manipulation to reduce alternate bearing and the need for often time-consuming tree management strategies.

## Fast-forwarding technologies towards precision tree crop production

Plant breeding has been transformed in recent years through the advent of rapid and precise genome-enabled breeding technologies. These cutting-edge methods have enabled breeding programs to accelerate genetic gain using techniques that accelerate the development of superior crop varieties (**Table 1**). In the following section we examine the spectrum of genome-enabled plant breeding methods. First, we delve into purely genomic techniques, such as MAS and genomic selection (GS). Both methods use the power of molecular markers and genomic data to identify and select plants with desirable traits more efficiently. By doing so, they can dramatically shorten breeding cycles and reduce the size of resource-intensive progeny trials. Moving beyond MAS and GS, we explore how advances in functional genomics and computational biology enable plant scientists to unravel the complex web of genetic interactions governing plant traits. Greater understanding of gene regulatory networks provides precise information into which genes are best to target for genetic modification, allowing molecular breeders to fine-tune desirable traits with precision. Finally, we review genetic manipulation in plants, evaluating the potential for transgenic and gene editing approaches to precisely modify traits in elite crop varieties. Most of these technologies are reliant on, or aided by, a sound understanding of the genetic basis of traits of interest, such as described in the previous sections.

**Table 1:** Selected examples of the use of different genomics approaches for trait improvement in several tree crops.

	Almond	Apple	Citrus	Mango
**Genomic selection (GS)**	Unknown	6 fruit quality traits in 1200 individuals from 6 parental cultivars (Kumar et al., 2012) 2 productivity and 8 fruit quality traits in 977 individuals from 24 parental cultivars (Muranty et al., 2015) 9 fruit quality traits and resistance to apple scab in 172 accessions (McClure et al., 2018) 30 quantitative traits related to phenology, productivity, fruit size, outer fruit, inner fruit and vigour in 269 accessions (Jung et al., 2022)	17 fruit quality traits in 111 citrus varieties (Minamikawa et al., 2017) Fruit weight, sugar content and acid content in 1935 individuals from 106 parental cultivars (Imai et al., 2019)	Trunk circumference, fruit blush colour and intensity in 108 mango accessions (Wilkinson et al., 2022)
**Marker assisted selection (MAS)**	Self-compatibility, leafing date, shell hardness, double kernel, productivity, blooming date, kernel weight, in-shell weight and kernel taste in ‘R1000’ and ‘Desmayo Largueta’ (Sánchez-Pérez et al., 2007) Resistance to root-knot nematodes in ‘Alnem’ and ‘Lauranne’ (Duval et al., 2018) Shell hardness in 'Nonpareil' and 'Lauranne' (Goonetilleke et al., 2018) and 264 almond accessions (Sideli et al., 2023). Kernel taste in 134 sweet kernel cultivars and 10 bitter kernel individuals (Lotti et al., 2023)	Scab, fire blight and powdery mildew resistance, fruit firmness, skin colour, flavour intensity and acidity in 207 accessions (Chagné et al., 2019) Resistance to apple root ring rot in ‘Jonathan’ and ‘Golden Delicious’ (Shen et al., 2019)	Disease resistance in trifoliate orange (Peng et al., 2020) HLB tolerance in ‘Argentina’ and ‘Flying Dragon’ trifoliate orange and ‘Sanford’ and ‘Succari’ sweet orange (Huang M. et al., 2023) Male sterility and polyembryony in ‘Kiyomi’ and ‘Murcott’ mandarin (Montalt et al., 2023)	Fruit weight in ‘Tommy Atkins’ x ‘Kensington Pride’ hybrids (Bally et al., 2021) Anthracnose resistance in 143 mango varieties (Felipe et al., 2022) Fruit colour and firmness in ‘Amrapali’/’Sensation’ hybrids (Srivastav et al., 2023)
**Transformation (overexpression)**	Unknown	Precocious flowering in ‘Pinova’ by overexpressing *MdFT1* (Tränkner et al., 2010) Reduced anthocyanin biosynthesis and red flesh colour in ‘Ballerina’ by overexpressing *MdHB1* (Jiang et al., 2017) Increased drought tolerance in ‘Royal Gala’ by overexpressing *MdDof54* (Chen et al., 2020) Delayed fruit ripening and increased fruit firmness in ‘Royal Gala’ fruit by overexpressing *ERF4* and *TPL4* (Hu et al., 2020) Reduced fruit firmness in ‘Golden Delicious’ fruit by overexpressing *MdACS3a* (Hu et al., 2022a) Increased resistance to osmotic stress and increased shoot and root growth in wild apple (*Malus sieversii*) by overexpressing *Msi-miR156ab* (Feng et al., 2023)	Precocious flowering in Carrizo citrange by overexpressing *AtLFY* or *AtAP1* (Peña et al., 2001) Increased carotenoid production and orange fruit colour in Hongkong kumquat by overexpressing *CsPSY* (Zhang et al., 2009) Enhanced salt stress tolerance in trifoliate orange by overexpressing *AhBADH* (Fu et al., 2011) Loss of thorns in Carrizo citrange by overexpressing *CsCEN* (Zhang et al., 2021) Enhanced tolerance to salt, oxidative stress, alkaline pH, drought and two pests in ‘Pramalini’ lime (*Citrus aurantifolia*) by overexpressing *PRpnp* (Singh et al., 2023) Enhanced resistance to CYVCV in ‘Eureka’ lemon by overexpressing *CIRPS9-2* (Zeng et al., 2023)	Unknown
**Transformation (RNAi)**	Unknown	Precocious flowering by silencing *MdTFL1* in ‘Pinova’ (Flachowsky et al., 2012) and ‘Orin’ (Kotoda et al., 2006) Reduced drought tolerance in ‘Royal Gala’ by silencing *MdDof54* (Chen et al., 2020) Increased drought tolerance, scion growth and photosynthesis in GL3 (‘Royal Gala’ progeny) by silencing six *MdGH3* genes (Jiang et al., 2022) Browning resistance in ‘Golden Delicious’, ‘Granny Smith’ and ‘Fuji’ by silencing *POO* (https://osfruits.com/science/biotech-information/)	Partial loss of thorns in Carrizo citrange by silencing *TI1* (Zhang et al., 2020)	Unknown
**VIGR**	Bleaching in ‘Nonpareil’ using ALSV to silence *PDS* (Kawai et al., 2016)	Precocious flowering in apple seedlings using ALSV to silence *MdTFL* (Sasaki et al., 2011) Precocious flowering in ‘Fuji’, ‘Orin’ and ‘Golden Delicious’ using ALSV to overexpress *AtFT* (Yamagishi et al., 2011) Induced fruit ripening in ‘Golden Delicious’ fruit using TRV to silence *MdERF2* (Li et al., 2016) Precocious flowering in ‘Ourin’ using ASLR to overexpress *AtFT* and silence *MdTFL1-1* (Yamagishi et al., 2016) Increased anthocyanin biosynthesis and red flesh colour in ‘Granny Smith’ using TRV to silence *MdHB1* and/or *MdMYB10* (Jiang et al., 2017) Increased ring rot resistance in ‘Fuji’ fruit using TRV to silence *MdCNGC2* (Zhou et al., 2020) Induced fruit ripening and reduced fruit firmness in ‘Gala’ fruit using TRV to silence *ERF4*, *TPL4* (Hu et al., 2020) or *JAZ* (Hu et al., 2022b) Reduced fruit firmness using TRV to silence *MdHDA19* in ‘Gala’ fruit and increased fruit firmness using TRV to silence *MdACS3a* in ‘Golden Delicious’ fruit (Hu et al., 2022a) Reduced ring rot resistance in ‘Granny Smith’ using TRV to silence *MdMAPKKK1* or *MdBSK1* (Wang N. et al., 2022)	Shorter height in *Citrus excelsa* and ‘Mexican’ lime using CLBV to silence *actin* (Agüero et al., 2014) Increased production of limonoids in ‘Guangxi Shatian You’ pummelo (*Citrus grandis*) using TRV to silence *CiOSC* (Wang et al., 2017) Bleaching in lemon, Changshou kumquat (*Fortunella obovata*), ‘Guangxi Shatian’ pummelo shoots using TRV to silence *CitChlI* (Wang F. et al., 2019) Altered citric acid levels in ‘Dafen-4’ and ‘Weizhang’ mandarin fruit using TRV to silence *CS* and *ACL* (Chen et al., 2023)	Fruit ripening and pericarp colouration in mango using TRV to silence *RCCR* (Liu et al., 2021) Fruit ripening and aroma in ‘Dashehari’ using TRV to silence *MiPMK* (Pathak et al., 2023)
**CRISPR gene editing**	Unknown	Precocious flowering in ‘Gala’ by editing *MdTFL1.1* (Charrier et al., 2019) Reduced fire blight susceptibility in ‘Gala’ and ‘Golden Delicious’ by editing *MdDIMP4* (Pompili et al., 2020) Increased ring rot resistance in ‘Fuji’ fruit by editing *MdCNGC2* (Zhou et al., 2020) Reduced ring rot resistance in ‘Orin’ by editing *MdMAPKKK1* (Wang N. et al., 2022)	Resistance to citrus canker by editing *CsLOB1* in ‘Duncan’ grapefruit (*Citrus* x *paradisi*; Jia et al., 2016a; Jia et al., 2017; Jia et al., 2022), ‘Wanjincheng’ sweet orange (Peng et al., 2017), and ‘Hamlin’ sweet orange (Huang et al., 2022; Su et al., 2023) Reduced susceptibility to citrus canker in ‘Wanjincheng’ orange by editing *CsWRKY22* (Wang Y. et al., 2019) Loss of thorns and increased branching in Carrizo citrange by editing *TI1* and/or *TI2* (Zhang et al., 2020) Conversion of axillary meristem into thorns in Carrizo citrange by editing *CsCEN* (Zhang et al., 2021) Decreased citric acid accumulation in Hongkong kumquat fruit by editing *CitPH4* (Huang Y. et al., 2023)	Unknown

### Genomics approaches

The improvement of horticultural tree crops has been modernised with the advent of high-throughput genomic technologies, i.e., high-throughput microarray genotyping and next generation sequencing, which have played pivotal roles in enhancing the precision, speed, and efficiency of tree breeding programs. These technologies and new selection methods have enabled tree breeders to accelerate genetic gain and the development of superior tree varieties with desirable trait combinations. High-throughput genotyping methods assay thousands of genetic markers simultaneously at a relatively modest cost and when analysed in segregating or reference populations have enabled the identification of key genetic variants associated with traits of interest, such as disease resistance, fruit quality, and plant architecture, among others (Peng et al., 2020; Wang L. et al., 2022; Huang M. et al., 2023; Srivastav et al., 2023). Comprehensive knowledge of the genetic basis of multiple agronomically important traits has allowed breeders to make informed decisions on the selection of parent trees for crossing in breeding programs, generating new hybrid varieties with improved trait combinations.

In tree species characterised by a long juvenile phase, traditional breeding methods can be slow and labour intensive. The use of MAS, which employs genetic markers closely associated with traits of interest, has emerged as a game-changer in this context. MAS allows breeders to screen large populations during the seedling stage and select those with the potential to exhibit the desired traits at maturity. For example, in citrus a hybrid population of ‘Kiyomi’ and ‘Murcott’ tangors segregating for male sterility and polyembryony was used to develop and validate markers tightly linked to both traits to be used for MAS in breeding programs (Montalt et al., 2023). In macadamia, the identification of markers tightly linked to nut quality characteristics could be used to screen for elite progeny potentially reducing the selection process by seven years in the breeding program (O’Connor et al., 2019). And examples in Rosaceae fruit trees include screening apricot seedlings for markers associated with plum pox resistance (Zuriaga et al., 2018; Polo-Oltra et al., 2020), apple seedlings for markers associated with fruit quality and disease resistance (Chagné et al., 2019; Petiteau et al., 2023), and peach seedlings for markers linked with fruit colour (Adami et al., 2013) increasing the efficiency of these breeding programs. This approach significantly reduces the time required for traditional phenotypic evaluation which for many traits can be cumbersome, thus accelerating the breeding cycle in tree crops. Moreover, MAS has the added benefit of eliminating individuals without specific alleles, streamlining the selection process and conserving resources by focusing only on candidates with the highest potential for success.

GS is another analysis method that relies on an alternative approach based on analysis of all QTL effects, regardless of their significance, which may improve tree breeding by increasing the accuracy and efficiency of variety selection. It enables breeders to focus their efforts on candidates with the highest genetic potential, resulting in more targeted and successful breeding outcomes. Unlike traditional breeding methods that rely on the phenotypic evaluation of individual trees, GS leverages comprehensive genomic data to predict the breeding value of trees based on their genetic makeup. GS has been applied with great success in cereal and livestock breeding (Xu et al., 2021) but is still in its infancy for horticultural trees. For example, a recent study in apple determined the genetic architecture of 30 important traits and set out a strategy to implement GS in apple breeding programs to improve selection strategies (Jung et al., 2022). Another example from macadamia demonstrated that using GS models could increase genetic gain for yield, which is more than double that achieved from traditional breeding (O’Connor et al., 2021). Consequently, GS has the potential to enable breeders to accurately estimate the performance of trees before they reach maturity, allowing for early identification of top-performing individuals.

### Regulation of crop trait genetic interactions

The recent and fast-paced advancements in high-throughput next generation sequencing technologies have also aided in the rapid increase of omics-based experiments to collect expression data. These transcriptomic data can be used to infer gene co-expression networks using a mathematical approach for transcriptomic data analysis resulting in gene co-expression network inference (Lee et al., 2015; Haque et al., 2019; Jiang et al., 2023). The network can be broken down into two components; nodes (genes and their products) represented by shapes, and edges representing interactions between the nodes. The network follows a guilt by association principle whereby genes that observe similar expression patterns are clustered together implying a potential (co)-regulatory relationship within the endogenous system (Lee et al., 2015; Haque et al., 2019). This approach is a relatively simple way to identify many of the genetic components underlying important agricultural traits and has already been implemented to study different traits for apple (Ding et al., 2021), peach (Khan et al., 2022), apricot (Yu et al., 2020), sweet cherry (*Prunus avium*; Yang H. et al., 2021), kiwifruit (Brian et al., 2021), and *Vitis* spp. (Toups et al., 2020; Tan et al., 2023). However, as co-expression network inference is an *in silico* inference method to identify genes of interest, these predictions need to be validated via experimental approaches (Haque et al., 2019) such as chromatin immunoprecipitation sequencing (ChIP-seq) (Robertson et al., 2007) and DNA affinity purification sequencing (DAP-seq) (O’Malley et al., 2016).

ChIP-seq is an *in vivo* technique first implemented on mammalian HeLa cells which utilises antibody-driven chromatin immunoprecipitation and DNA sequencing to identify direct genetic targets of transcription factors via read mapping against a reference genome (Robertson et al., 2007). The potential of ChIP-seq was further expanded to identify histone modifications in the genome landscape which are correlated with gene expression changes (Pillai et al., 2009). ChIP-seq has since been used for studying both the targets of transcription factors as well as the genome-wide patterns of histone modifications underlying traits such as fruit ripening, dormancy, and stress-response in various horticulture crops including peach (de la Fuente et al., 2015; Canton et al., 2022; Jin et al., 2022; Zhao et al., 2023), sweet cherry (Vimont et al., 2019), kiwifruit (Wu et al., 2018), and apple (Chen et al., 2020; Hu et al., 2020; Chen et al., 2022; Hu et al., 2022a; Hu et al., 2022b).

In contrast to ChIP-seq which requires costly antibodies and is not easily upscaled, an alternative *in vitro* method called DAP-seq was created by O’Malley et al. (2016). The technique uses an affinity tagged transcription factor to bind genomic DNA and identify downstream target genes via DNA sequence read mapping. The number of regulators can be easily upscaled in this method, with O’Malley et al. (2016) using DAP-seq on 1812 Arabidopsis transcription factors and the maize ZmARF29 transcription factor. DAP-seq has since been used in numerous horticultural perennial crops including apple (Chen et al., 2020; Falavigna et al., 2021), sweet orange (Wang W. et al., 2023), grapevine (*Vitis vinifera*; Orduña et al., 2022) and avocado (Núñez-Lillo et al., 2023) to study traits like flowering, fruit softening, starch synthesis, disease resistance, and stress-induced responses.

Network inference is a promising tool for molecular breeding as it allows the use of transcriptomic data to confirm the association of genes previously identified through functional genomics experiments with traits of interest, and associate new genes with these traits. Furthermore, it can allow us to identify potential interactions between previously identified and newly identified genes for validation via techniques like ChIP-seq and DAP-seq. Finally, network inference can assist with selecting the best candidates for manipulation of single traits.

### In planta bioassays – leveraging knowledge from trees in the field for selecting seedlings with desirable traits

Plants have evolved to synchronise flowering with favourable environmental conditions to ensure successful reproduction (Kim et al., 2009). The ability of plants to flower in response to the correct environmental stimuli is gained during either the vegetative or reproductive phases of organ development. The prolonged juvenile phase of perennials makes it difficult to unravel molecular mechanisms controlling reproductive organ development and floral initiation in the early years of perennial growth, and to assess horticultural traits such as fruit quality can take years.

There is a need for more creative approaches by comparing gene regulatory processes in different tissues from trees growing in the field with those growing in an environmentally controlled growth environment. For example, by examining transcriptomic profiles in field grown tree tissues responding to a decline in photoperiod, cold snaps, and progressive chilling period, it would be possible to untangle the gene regulatory networks sensing, responding, and modulating phases of floral bud dormancy and the hence the onset of flowering. An integrated approach that evaluates gene regulatory processes in source tissues such as the leaves and sink tissues such as floral buds could enable predictions of the key events modulating early to late cultivar floral initiation. By simulating these climate events within a controlled environment, it might be possible to replicate the transcriptomic networks of gene regulations within seedling tissues thereby enabling the fast-forwarding screening of individuals in a segregating population that perform according to the tree growing in the field.

This approach could be routinely established by also combining external chemical treatments to the seedlings growing in a controlled environment to further simulate the use of plant growth regulators used within field conditions to alter gene regulatory networks that facilitate flowering. The role of phytohormones and their crosstalk with environmental signals during flowering in annuals is well studied (Gerashchenkov and Rozhnova, 2013), but less so in perennials due to the long juvenile period. Mølmann et al. (2005) experimented with juvenile hybrid aspen by treating with paclobutrazol combined with low night temperature and short-day conditions. As expected, paclobutrazol reduced GA and caused growth cessation. It also promoted bud set and improved cold hardiness, a trait that is important for perennials to survive harsh environmental conditions during endodormancy. However, it did not promote dormancy suggesting that the ABA pathway can maintain dormancy independent of GA. The application of omics approaches to quantify hormones, metabolites, and gene expression in tissues from trees and seedlings grown in controlled environments, could fast-track knowledge of how the environment modulates dormancy and flowering. Ultimately, devising a tree to seedling bioassay could improve the development of new molecular markers to screen for important tree crop traits and reduce the number of trees from segregating populations to be planted in the field.

### Functional genetics for trait characterisation and improvement

The development of the first transgenic plant in 1983 (Herrera-Estrella et al., 1983) gave scientists and plant breeders high hopes that transgenic technology would revolutionise our understanding of plant molecular biology and fast-track crop breeding. However, the negative public perception and intense biosecurity regulation did not allow transgenic technology to be used to its full potential. A prime example of this was the development of transgenic American chestnut (*Castanea dentata*) which is resistant to fungal blight disease (Powell et al., 2019). Blight disease has destroyed billions of wild-type American chestnuts, yet transgenic plants with resistance to this disease have not yet been deregulated for public use. Nevertheless, it must also be noted that the regulations restricting genetically modified organisms (GMOs) are slowly being relaxed in different countries. This has resulted in a steady increase in the total acreage under GM crop cultivation in the last decade (Lobato-Gómez et al., 2021). In Australia, commercial cultivation of four genetically-modified crops: canola (*Brassica napus*), mustard (*Brassica juncea*), cotton (*Gossypium hirsutum*) and safflower (*Carthamus tinctorius*) has been approved (OGTR, 2023). Globally, different transgenic crop cultivars including several horticultural crops such as apple, plum, papaya (*Carica papaya*), eggplant (*Solanum melongena*), sweet pepper (*Capsicum frutescens*) and watermelon (*Citrullus lanatus*) have been approved for commercial cultivation (Baranski et al., 2019). These GM crops have improved agronomic traits such as fruit browning in apple (Waltz, 2015), resistance to insects in eggplant (Ahmed et al., 2021), and resistance to viral disease in plum (Singh et al., 2021), papaya (Gonsalves et al., 2007), watermelon (Lin et al., 2012) and sweet pepper (Chen et al., 2003). The future of GM crops is encouraging since many crop cultivars are in the pipeline for deregulation, and public perception and legalities are changing around the world with many countries slowly beginning to embrace GM crops.

Transgenic technologies have provided alternatives to employing forward genetics in horticultural trees. Predicting gene functions using forward genetics requires developing mapping populations which is time-consuming and resource-intensive due to the long juvenile phase of trees and resources needed for maintaining a large number of trees. Transgenic technologies in addition to the public availability of genome databases of different horticultural trees and along with the progress in the development of different tools for phylogenetic analysis have simplified gene function studies in trees. These techniques have been used to identify homologs of genes from model plants in tree crops and transgenically overexpress those genes either in Arabidopsis or in the trees themselves. These techniques have been used in several tree crops for studying genes related to different biological processes, including those already described in this review such as tree architecture, bud dormancy and flowering time. In addition to these processes, transgenic approaches have also been used to study fruit quality, disease resistance and other traits of economic significance. For example, in Carrizo citrange, overexpression of Thionin increased resistance to citrus canker and Huanglongbing disease in transgenic citrus plants (Hao et al., 2016). While transgenic overexpression of peach NAC25 in Nanlin895 poplar (*Populus* x *euramericana*) shoots increased anthocyanin biosynthesis and transport resulting in redder shoot tips, indicating that NAC25 could play an important role in red fruit colour development in peach (Geng et al., 2022). Results from these and other studies have shown that gene function knowledge from model plants can be exploited using transgenic technologies to bridge the gap in functional genomic studies between model plants and tree crops.

In contrast to transgenic overexpression where the gain-of-function strategy is utilised to characterise gene functions, another transgenic tool called RNAi utilises a loss-of-function strategy to characterise gene functions. RNAi induced post-transcriptional gene silencing has been used to characterise genes related to different biological pathways in transgenic plants (Small, 2007). For example, in apple three *DAM* and two *SVP* genes were silenced simultaneously resulting in the development of an evergrowing phenotype (Wu et al., 2021) similar to that observed in the natural *evergrowing* peach mutant (Bielenberg et al., 2008). While in transgenic strawberries (*Fragaria x ananassa*), an RNAi construct designed using a jasmonic acid biosynthetic gene *ALLENE OXIDE SYNTHASE* (*AOS*) from grapevine triggered silencing of *FaAOS* and caused an un-colouring phenotype in strawberry fruit (Jia et al., 2016b). Apart from gene function studies, RNAi-based gene silencing has also been used for improving cultivars and rootstocks. One of the first RNAi-based transgenic commercial cultivars was developed in papaya to protect against Papaya ringspot virus (PRV; Ferreira et al., 2002). A similar strategy was used in developing the plum ‘Honeysweet’ cultivar, which contains an RNAi construct with plum pox virus coat protein-derived hairpin and is resistant to plum pox virus (Scorza et al., 1994). Apple cultivar Arctic^®^, grown by Okanagan Specialty Fruit Inc., is another RNAi-based cultivar with a browning resistance phenotype (https://osfruits.com/science/biotech-information/). Meanwhile, RNAi-induced knockdown of six apple *GH3* genes increased drought tolerance in apple rootstock and increased scion growth and photosynthesis (Jiang et al., 2022). And in a sour cherry (*Prunus cerasus* x *P. canescens*) rootstock, expression of an RNAi construct containing a Prunus necrotic ringspot virus (PNRSV) RNA3 hairpin caused resistance to PNRSV in wild-type scion grafted on transgenic rootstock (Zhao and Song, 2014). These findings highlight the potential of RNAi in both gene-function studies and crop improvement.

Transgenic technologies have inherent limitations in fruit trees. For example, developing transgenic plants requires a long time and many fruit tree species are recalcitrant to tissue culture required for development of transgenic plants (Lobato-Gómez et al., 2021). These limitations can be overcome by using a virus-induced gene regulation (VIGR) system. VIGR technology exploits the ability of viruses to multiply in plants and the plant antiviral defence system to overexpress (virus-induced gene overexpression (VIGO)) and silence (VIGS) target genes in plants (Paudel et al., 2022). Tobacco rattle virus (TRV) and apple latent spherical virus (ALSV) based VIGR systems are two of the most common systems used for VIGS and VIGO in fruit trees such as peach, apple, European pear, Japanese pear, almond and mango (Paudel et al., 2022; Pathak et al., 2023). In fruit trees, VIGR systems have been mostly used for functional genomics to characterise genes regulating different biological pathways such as carotenoid accumulation and fruit ripening in peach (Li et al., 2017) and mango (Pathak et al., 2023), pathogen resistance in peach (Cui and Wang, 2017), and flowering in apple, citrus, Japanese pear and *Vitis* spp. (Velázquez et al., 2016; Yamagishi et al., 2016; Maeda et al., 2020). Apart from functional genomics studies, VIGR can also be used to reduce the juvenile phase in fruit trees for fast-track tree breeding (**Figure 4**). This approach was used in apple to select a fire blight-resistant apple genotype in fifth generation plants within seven years (Schlathölter et al., 2018). Considering that the juvenile phase of apple lasts for 5-12 years, this process would have taken several decades using traditional breeding methods.

However, there are also limitations to VIGR, including a limited host range of the viruses used (Silva et al., 2010), non-uniform gene silencing (Burch-Smith et al., 2004), and immunogenicity and unintended mutations in the host (Li et al., 2018). The use of nanoparticles to deliver RNA for gene silencing or plasmid DNA for gene expression may overcome some of these issues. One advantage of nanoparticles is their small size ranging from 1-100 nm in at least one dimension (Cunningham et al., 2018). As such nanoparticles can be designed to fit within the size exclusion limit of cell wall pores, usually ~20 nm (Kumar et al., 2020) allowing for the application of nanoparticles to potentially any plant species. Larger nanoparticles can also be modified to cause structural changes to the cell wall to allow their entry (Kumar et al., 2020), which is beneficial for delivering larger cargo such as plasmid DNA. Modifications to nanoparticles can also allow for targeted subcellular localisation such as to the chloroplast or mitochondria (Yoshizumi et al., 2018; Thagun et al., 2022). Furthermore, nanoparticles can deliver a wide range of cargo to the cell, including DNA, RNA, protein, plant hormones and chemicals such as pesticides and fertilisers (Kumar et al., 2020; Hu and Xianyu, 2021). While nanoparticles have been tested often in model species e.g. to deliver dsRNA or siRNA for gene silencing (Jiang et al., 2014; Demirer et al., 2019; Schwartz et al., 2020; Yong et al., 2022), plasmid DNA for transient overexpression (Chang et al., 2013; Demirer et al., 2019) or stable transformation (Burlaka et al., 2015; Zhao et al., 2017), or protein for stable transformation (Martin-Ortigosa et al., 2014), limited studies have been done in tree crops. Lipid-based nanovectors were used to deliver the phytohormones indole-3-butyric acid (IBA) and 1-Naphthaleneacetic acid (NAA) to olive trees to improve the rooting process both *in vitro* and *in vivo* (Clemente et al., 2018). And a recent study by Qiao et al. (2023) used nanovesicles for dsRNA delivery to induce gene silencing for crop protection against a fungal pathogen in grapevine, fox grape (*Vitis labrusca*), rose (*Rosa hybrida*), lettuce (*Lactuca sativa*) and tomato, demonstrating the applicability of this technology in tree crops and other perennial herbaceous crops.

### Engineering new varieties by gene editing

Since the discovery of the Nobel prize winning CRISPR-Cas9 system by Jinek et al. (2012), the use of CRISPR-Cas9 and other Cas proteins to perform directed changes to the genome has dominated the field of genetic engineering. The benefits of this system are the precise manner in which these changes can be made, the potential for the technology to be applied to theoretically any organism, and the potential for engineering non-GM gene edited varieties. In fact, some CRISPR-edited crops have already been released such as ɣ-aminobutyric acid (GABA)-enriched tomatoes with improved nutritional profiles (Ezura, 2022) and mustard salad greens with loss-of-function myrosinase genes to improve the taste profile by eliminating pungency (Karlson et al., 2022). Several examples of CRISPR-edited tree and woody crops also exist (e.g. apple (Charrier et al., 2019), kiwifruit (Herath et al., 2022), Hongkong kumquat (*Citrus hindsii*; Zhu et al., 2019), sweet orange (Jia and Wang, 2014) and avocado (https://www.prnewswire.com/news-releases/ag-biotech-innovator-greenvenus-achieves-breakthrough-in-non-browning-avocado-through-gene-editing-301842939.html), although none have been commercially released yet, and many tree crops like macadamia and mango have no published examples of CRISPR-editing. Despite the successful use of CRISPR technologies in several tree crops, many challenges remain.

One challenge faced with CRISPR technologies is the transfection step. *Agrobacterium* is commonly used as the transfection vector for the CRISPR-Cas transgene, however, transfection rates are lower for differentiated tissue (e.g. epicotyl) compared to cell-based cultures (Dutt et al., 2018) and many species are not amenable to transfection by *Agrobacterium* (Song et al., 2019). The use of ribonucleoproteins (RNPs) (Mout et al., 2017b; Zhang et al., 2022) and/or nanoparticles (Mout et al., 2017a) to deliver CRISPR-Cas products may be one solution for species not amenable to *Agrobacterium*-mediated transformation. The use of cell-based cultures can also reduce the risk of chimerism, an issue often encountered when tissues such as epicotyls are transfected, however, this does not eliminate the risk completely (Sahijram and Bahadur, 2015). Chimerism may also be reduced through multiple rounds of shoot regeneration, as shown by Ding et al. (2020) in ‘Shanxin’ poplar (*Populus davidiana* × *P. bolleana*) trees. These methods also all require tissue culture to regenerate gene edited plants, which itself is a challenge in many tree crops. Recent innovative methods have successfully bypassed the tissue culture process by overexpressing developmental regulators to stimulate organogenesis in somatic cells generating gene edited plantlets within 2-4 weeks in maize (Lowe et al., 2018), Arabidopsis, *Nicotiana benthamiana* (Maher et al., 2020), and sorghum (*Sorghum bicolor*; Che et al., 2022). Another issue with many of these methods is the requirement for the insertion of transgenes expressing the CRISPR components. This means that the T1 generation is transgenic, and conventional breeding is required to select out the transgene. Yang L. et al. (2023) demonstrated a method to create a transgene-free T1 generation using rootstocks expressing mobile Cas9 mRNA and gRNA to create gene edited Arabidopsis and *Brassica rapa*. Mendelian genetics potentially allows for a transgene free T1 generation that carries a gene edit, even with a transgenic T0 generation, providing the transgene and gene edited allele are not linked. Cross-pollination allows for the T1 generation to be added to the conventional breeding pipeline, thus complementing, and not replacing known plant breeding techniques. However, the use of many of these advances in CRISPR have not yet been demonstrated in tree crops.

Another remaining challenge is the issue of chromatin accessibility influencing gene editing efficiency as chromatin can impede the Cas9-gRNA complex accessing the target sequence. Weiss et al. (2022) showed that methods to study chromatin accessibility, like ChIP-seq (by targeting histones associated with closed chromatin), Formaldehyde-Assisted Isolation of Regulatory Elements (FAIRE)-seq, and Assay for Transposase-Accessible Chromatin (ATAC)-seq, may also provide directions in improving editing efficiency of certain genes. Since gene expression also varies over time and per tissue type, gene expression data can also be leveraged to understand what tissues to target and when. Combining gene expression data with chromatin accessibility could also provide more defined targeting strategies.

The CRISPR system itself also has potential for improvement through methods like base-editing (Komor et al., 2016) and prime-editing (Anzalone et al., 2019). Both methods improve upon the standard CRISPR-Cas9 mechanism of inducing double-strand DNA breaks (DSBs) and relying on endogenous DNA repair pathway machinery to introduce errors, by inducing specific base pair edits without requiring DSBs. Base-editing utilises a Cas9 fused with a deaminase allowing for transition and some transversion mutations to occur without DSBs, while prime-editing utilises a Cas9 nickase fused with a reverse transcriptase variant to create a single-strand DNA break which, along with a guide RNA containing an edited RNA template, can introduce both transition and transversion mutations, as well as small insertion or deletion mutations (Chen and Liu, 2023). Although both base-editing and prime-editing have been demonstrated in several plant species (Azameti and Dauda, 2021), its use in plants is still in early stages and requires improvement to be viable for commercial breeding. Other improvements include the use of different Cas proteins, like Cpf1/Cas12a (Zetsche et al., 2015; Zhang et al., 2022) with different recognition sites.

Even if the science of CRISPR-Cas shows potential, challenges remain on the regulatory end. CRISPR-Cas9 is a patented system with Corteva and MIT, Broad Institute holding the licensing rights (Corteva Agriscience, n.d.), which means royalties from products using the technology must be considered. Whether CRISPR-edited crops are considered a GMO differs around the world. Jurisdictions like New Zealand (Kershen, 2015) and the European Union (EU; Court of Justice of the European Union (CJEU), 2018) regulate all CRISPR gene edited plants as GMOs. While in Australia, a CRISPR gene edited organism is not considered as a GMO if no foreign genetic material is present (Mallapaty, 2019), though this must be proven to regulators using techniques like genome sequencing. The legal landscape is also constantly evolving with the EU recently requesting for comment changes to the regulations surrounding gene editing (Laaninen, 2021; Mehta, 2023). Additionally, most CRISPR gene edited plants involve the insertion of a transgene in the T1 generation, making them GMO in many jurisdictions, and requiring multiple rounds of breeding to select out the transgene. The European Patent Office also decided a case in 1999 where a key decision stated that ‘plant varieties containing genes introduced into an ancestral plant by recombinant gene technology are excluded from patentability’ (European Patent Office, 1999, p.35). By extension, this decision likely applies to CRISPR-edited plants which are seen as GMOs in the EU further reducing incentives for using gene editing in the EU agricultural sector. Despite these legal challenges, CRISPR has opened multiple possibilities for improving tree breeding. However, the many limitations associated with the use of CRISPR in trees, along with regulatory hurdles mean that CRISPR-edited tree products will likely not get to the market immediately.

## Concluding remarks

The rich history of traditional horticultural tree breeding has domesticated a diverse variety of fruits, refined through generations of crossing and selection. This process has generated high-yielding citrus, apple, almond and avocado cultivars, among others, that have become the staple fruits produced in orchards around the world. Yet, in the face of evolving challenges and opportunities posed by shifting climatic patterns, the growing demand for food and the emergence of automation, the limitations of conventional breeding methods are becoming increasingly apparent. To effectively address these threats to horticultural production and to rapidly develop resilient varieties, there is a need for greater integration of genome enabled technologies into horticultural tree breeding programs. Apple breeding is one of several industries that has led the way for integration, showcasing remarkable success in expediting the development of novel cultivars boasting enhanced disease resistance, drought resilience, and superior fruit quality. The advent of low-cost, large-scale genome sequencing and the expanding repertoire of genomic approaches, such as those outlined in this review, are shifting the research and development landscape in tree crop species enabling greater integration of genome-enabled technologies across the horticultural tree crop breeding sector. By embracing genome enabled technologies in traditional breeding programs, we not only increase our capacity to develop new varieties, but also produce tree crops better suited to existing and emerging challenges, safeguarding the future of next generation orchards.

## Author contributions

SK: Conceptualization, Writing – original draft, Writing – review & editing, Visualization. SS: Writing – original draft, Writing – review & editing. LP: Writing – original draft, Writing – review & editing, Visualization. MM: Writing – original draft, Writing – review & editing. LS: Writing – original draft, Writing – review & editing. AJ: Writing – original draft. JV: Writing – original draft, Writing – review & editing. MT: Writing – review & editing. CC: Conceptualization, Writing – review & editing, Writing – original draft. EV-G: Conceptualization, Writing – original draft, Writing – review & editing, Visualization. PP: Writing – original draft, Writing – review & editing.
